# Development of a Control System for the Teat-End Vacuum in Individual Quarter Milking Systems

**DOI:** 10.3390/s130607633

**Published:** 2013-06-13

**Authors:** Ulrich Ströbel, Sandra Rose-Meierhöfer, Hülya Öz, Reiner Brunsch

**Affiliations:** 1 Leibniz Institute for Agricultural Engineering Potsdam-Bornim (ATB), 14469 Potsdam, Max-Eyth-Allee 100, Germany; E-Mails: srose@atb-potsdam.de (S.R.-M.); rbrunsch@atb-potsdam.de (R.B.); 2 Ege Vocational Training School, Ege University, Bornova, Izmir 35100, Turkey; E-Mail: hulya.oz@ege.edu.tr

**Keywords:** vacuum control valve, pressure sensor, vacuum control system, fluctuation, reduction, milk flow rate, teat-end

## Abstract

Progress in sensor technique and electronics has led to a decrease in the costs of electronic and sensor components. In modern dairy farms, having udders in good condition, a lower frequency of udder disease and an extended service life of dairy cows will help ensure competitiveness. The objective of this study was to develop a teat-end vacuum control system with individual quarter actor reaction. Based on a review of the literature, this system is assumed to protect the teat tissue. It reduces the mean teat-end vacuum in the maximum vacuum phase (b) to a level of 20 kPa at a flow rate of 0.25 L/min per quarter. At flow rates higher than 1.50 L/min per quarter, the teat-end vacuum can be controlled to a level of 30 kPa, because in this case it is desirable to have a higher vacuum for the transportation of the milk to the receiver. With this system it is possible for the first time to supply the teat end with low vacuum at low flow rates and with higher vacuum at increasing flow rates in a continuous process with a three second reaction-rate on individual quarter level. This system is completely automated.

## 1. Introduction

Many milking systems are equipped with sensors for recording data. For monitoring and control of livestock production processes, the precision livestock farming (PLF) approach makes use of modern monitoring and control theory [1]. However, despite PLF, currently available milking systems usually do not have a unit for acquiring and storing the teat-end vacuum data up to now. Further, to measure both the milk flow accurately for a reaction in a three second interval and the individual quarter milk-flow in the milk tube represents a substantial challenge. Despite the importance of automated measured milk flow data, little effort has been made to monitor the reliability of recording equipment on a daily basis [2]. The milk-air mixture in the milk tube excludes methods of measurement which have been developed for homogeneous liquids in pipelines.

Principally, the supply of steady vacuum conditions in a milking system is one of the most important preconditions in ensuring satisfactory system performance [3]. Under performance the authors in [3] understand characteristics such as situation of stimulation, state of bimodalities, and state of decline phase in the milk flow curve, state of overmilking, milk yield and level of somatic cell count (SCC). Nordegren [4], Schlaiß [5] and Spohr *et al.* [6] came to the opinion that cyclic vacuum fluctuations have no negative effect on the udder health, but that the risk of udder infection increases at the end of the milking process, when very low milk flows (and overmilking) occur. With the measurement and calculation of the teat-end vacuum reduction and fluctuation for b-and d-phase a more exact interpretation of the data is possible and will help estimate the effect on the udder health.

Further, Rasmussen *et al.* [7] observed high fluctuations and problems with vacuum stability in a conventional milking system with long milk tubes, when the air-inlet had a too small dimension. Vacuum fluctuations can have a negative impact on teat tissue [3]. The individual quarter, teat-end vacuum data can be evaluated for controlling the vacuum at this point during the entire milking process [8]. A more sophisticated technique for combining the evaluation and the control of teat-end vacuum would be desirable for the future of the milking technology. Then, it is possible to evaluate whether the teat-end vacuum is within the limits tested by Rasmussen and Madsen [9] for low vacuum (26 to 30 kPa on average) or high vacuum (33 to 39 kPa) at the teat end in that study. Negative changes of the teat-ends and in the teat canal are caused by mechanical forces during machine milking. The magnitude of the force depends on milking and pulsation vacuum, machine-on-time (mot), liner type and teat size [10–14]. In addition, a teat-end in good condition is the primary physical barrier in resisting bacterial colonization of the mammary gland (for example, with mastitis pathogens) [10,15].

The length and diameter of the teat canal make it susceptible to the entry and accumulation of bacteria and pathogens [16]. One example for negative changes in teat tissue is hyperkeratosis. It has a negative impact on the defence reaction of the teat. In the fissured keratin rings of hyperkeratosis, an enhanced bacterial colonization was observed, but no significant correlation between hyperkeratosis and mastitis infections was found by some studies [15,17,18]. However, other studies found the opposite, that teat-end callosities were predictive of clinical mastitis [19,20]. Therefore, a treatment that does not traumatize the teat-ends during the milking process, by adapting the vacuum level to each udder quarter, is necessary. Hamann *et al.* [21] showed that a milking system with positive pressure supply to the teats caused significantly smaller teat-end diameters and lower thickness values as compared to a conventional system.

Sagkob *et al.* [22] also compared a conventional milking cluster with a cluster which produces low teat-end vacuum in the minimum vacuum phase (d) of the pulsation cycle. From this study it was concluded that the low teat-end vacuum in the d-phase leads to a significant reduction of hyperkeratosis and teat-rings located around the teat-base on the evaluated udders.

Thus, the optimal teat-end vacuum curve should show a vacuum between 26 to 39 kPa [9] in the maximum vacuum phase (b) and a much lower and steady teat-end vacuum in the minimum vacuum phase (d). During the complete time-span of the b-phase, the vacuum should be as constant as possible. A study by Ipema and Hogewerf [23] revealed noticeable differences in milk-flow profiles of cows per udder quarter. For optimal milking, the process control of the milking system should take this into account for a milk-flow controlled milking. High teat-end vacuum (33 to 39 kPa) [9], should only be adjusted when the milk flow is higher than 0.8 L/min per quarter, because only then the high vacuum is required also for the transportation of the milk, resulting in more efficient milking.

The motivation of the present study is the assumption that, in the future, the number of lesions, oedema and hyperkeratosis can be reduced through the application of a more appropriate teat-end vacuum and teat-end massage during milking.

From this perspective, the objective of this study was to gather the data for constructing the hard-and software of a prototype for the proposed individual quarter control system. The hypothesis of the study is that it is possible to produce low teat-end vacuum at low and high vacuum at high milk flow rates and more general that the control system can produce vacuum conditions as stated in the description of the optimal vacuum curve.

## 2. Materials and Methods

### 2.1. Test Set-Up

In the experiments conducted, the effect of the vacuum control valve's (VCVs) settings and the effect of the flow rate on the vacuum reductions at the end of an artificial teat in an individual quarter milking system were measured. The wet-test-method, according to ISO 6690 (2007) [24], was conducted in a laboratory milking parlour. The parlour was a herringbone parlour with low line installation. The distance between milkline and floor of the parlour was 300 mm. The wet-test-method was conducted with four ISO artificial teats, built according to ISO 6690 [24]. Water at room temperature of 20 °C was used to simulate the effects of the milk flow [24]. In order to simulate “the milk flow by water”, four flow meters (Parker Hannifin Corporation, Cleveland, OH, USA) were used. During all the experiments the liquid flow rates at all four flow meters were set to the same level at all four udder quarters. In the wet-tests the vacuum was measured using a MilkoTest MT52 measuring system (System Happel^®^ GmbH, Friesenried, Germany) sampling with 500 Hz, with an accuracy of ±0.1 kPa, while the measuring accuracy of ±0.6 kPa was required in ISO 6690 [24].

The vacuum at the end of the artificial teat (connection with a 40/3 mm tube from the metal tube on the teat to the external Sensor 3 of MT52), in the pulse chamber and in the main vacuum line, was measured. Vacuum Sensor 1 of MT52 was connected to the machine vacuum (connection with a 2000/8 mm tube between internal Sensor 1 and the measuring point for the machine vacuum). Sensor 2 was connected to the pulse chamber through a T-piece. This was placed at a distance of 30 mm under the end of the teat cup in the short pulse tube (connection with a 200/8 mm tube). The T-pieces are well-suited for laboratory measurements [25].

**Figure 1.**
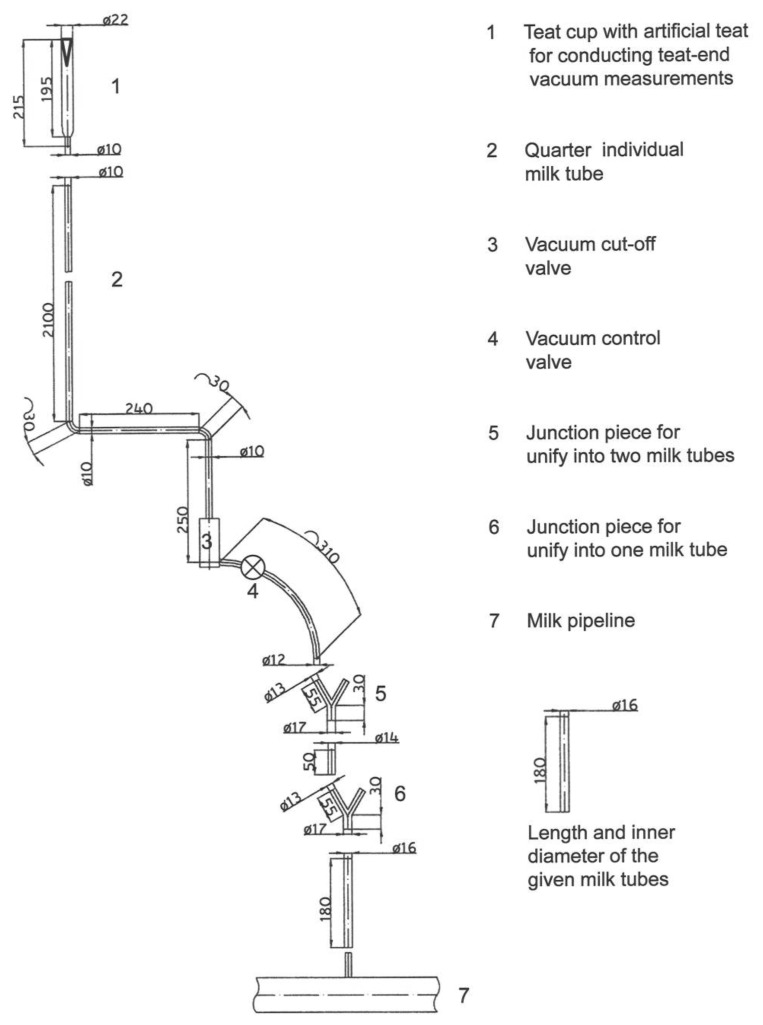
Test set-up with Multilactor^®^ (MULTI), vacuum control valve (VCV) and the artificial teat [26].

In detail, the wet-test-method was conducted with the following adjusted liquid flow rates at the four flow meters: 0.00, 0.25, 0.38, 0.50, 0.63, 0.75, 0.88, 1.00 and 1.13 L/min per quarter. The experiments were conducted in the single tube milking system Multilactor^®^ (MULTI). Figure 1 shows the pipe and tube length and the inner diameters of the duct system in MULTI, including the vacuum control valve (VCV) and the artificial teat [24]. Three quarters were not equipped with VCV in this series of tests, only for the rear left quarter a VCV was added to the test set-up. Nevertheless, during the wet-tests the other three quarters were also in operation and connected to artificial teats.

The MULTI is equipped with the sequential pulsation and with one vacuum cut-off valves in each of the four milk tubes. The valves react promptly to unwanted teat cup fall-offs. Sequential pulsation enables even milk flow and reduces vacuum reductions in the maximum vacuum phase (b-phase) of the pulsation cycle, in comparison to simultaneous pulsation, when different pulsation settings were tested in MULTI [27].

Further technical details of the MULTI, which were constant during all experiments, are given as follows: the year of production was 2009, the machine vacuum was 35 kPa, the pulsator ratio was 65% and the pulsator rate was 60 cycles/min. The tube length from the teat cup to the junction point was 3,095 mm and the inner tube diameter was 10 mm. MULTI was equipped with silicone liners. The compressive force to collapse the liner was 14 kPa for each liner.

The Biomilker^®^ technology was further integrated to MULTI and it includes periodic air inlet under each teat, when the liner is closed at the end of each pulsation cycle [28]. The reason for using MULTI in combination with the Biomilker^®^ technology was that MULTI was the only system which offers individual quarter milking in milking parlours, when the research work was started in 2008 and the aim was to control the vacuum on individual quarter level regarding the milk flow. On the other hand the whole construction of the MULTI-system is prepared for the Biomilker^®^ technology, MULTI for example is constructed with relatively long and low inner diameter milk tubes. The Biomilker^®^ helps to avoid high vacuum reductions in b-phase in the given construction. The air-flow through the Biomilker^®^ was 16.5 L/min for one teat cup (the situation without air consumption at all the clusters in the parlour was compared with milking of four teat cups of MULTI at a pulsator rate of 60 cycles/min and a pulsator ration of 60% with a liquid flow rate of 0.0 L/min per quarter at a machine vacuum of 36 kPa).

**Figure 2.**
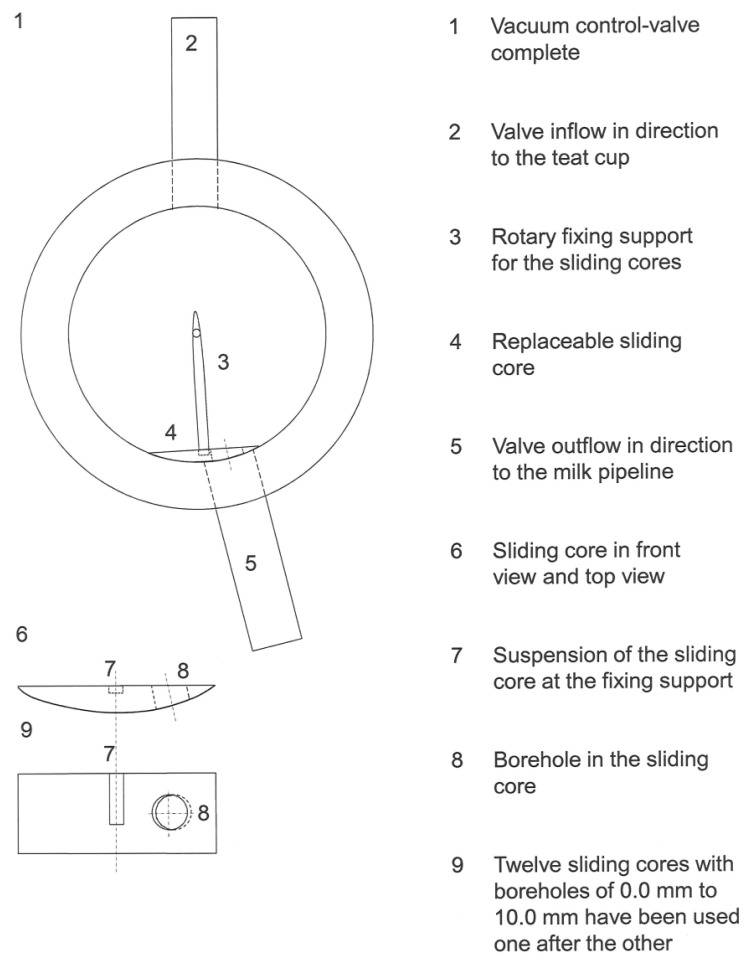
A drawing of the vacuum control valve (VCV), tosgether with an example for the twelve sliding cores, which were used for the tests [26].

The vacuum control valve (VCV) is shown in Figure 2. The applicable sliding cores of the VCV are enabled in order to simulate different VCV settings. The measurements took place in a factorial-design that involved twelve VCV settings and nine different liquid flow rates. In order to adjust the opening space of the valve's outflow, twelve sliding cores were produced which have the following boreholes: 0.0, 1.2, 1.5, 2.0, 2.5, 3.0, 4.0, 5.0, 6.0, 7.0, 8.0 and 10.0 mm. Each sliding core can be attached to the rotary fixing support. The sliding core has been exchanged for each new test series. The sliding cores could be exchanged, while the VCV is equipped with a visible and detachable cover of acrylic glass.

### 2.2. Calculation of Characteristic Vacuum Curves

Based on the data obtained, the mean teat-end vacuum and the vacuum reduction in b- and d-phase, along with the percentual time share of the phases of the pulsation cycle were calculated [24]. In total, the data of five pulsation cycles and eight repetitions of each test were used for calculating the mean vacuum data at each particular test [24]. The vacuum reductions were calculated as the difference between the machine vacuum and the mean teat-end vacuum out of forty cycles for each b- and d-phase. The determination of pulsation phases was carried out using a customised SAS macro (SAS Institute, Cary, NC, USA) according to the formulae presented in ISO 3918 (2007, paragraph 2.7.2) [29] and 6690 (2007, paragraph 8.2) [24]. With this procedure it was possible to calculate the “characteristic curves” for each flow rate, depending on the borehole diameter of the sliding core used. More precisely, the mean vacuum for each borehole diameter used for the b- or d-phase was computed out of the eight repetitions. These repetitions were measured at each combination of flow rate and sliding core.

### 2.3. Development of Control Models

With the help of the characteristic curve diagrams, pointed at Figures 4 and 5, and with the knowledge gleaned from the literature review [3,6,7,21,22], it was possible to work out three different control models in this study (*cf*. see Introduction). All the data for the three different control models, which are adjustable with the help of a VCV, were selected to meet the requirements of afore mentioned optimal teat-end vacuum curve in b-phase. The optimal teat-end vacuum curve in d-phase is not researched or confirmed. However, with the developed vacuum control system and the completely developed VCV, the vacuum can be optimised in a farm test.

One mechanical restriction of the electric vacuum control valve models is that it is impossible currently, to have different settings in the b-and d-phase within one pulse cycle, using the currently tested VCV and software, because the software reacts in half second rate. Thus, an optimal model function has to be found which fits for both decisive pulse phases (b- and d-phase). The previously calculated characteristic curves include all the teat-end vacuum data that was produced with the newly developed system. Thus, the objectives for the development of the three models as stated above are as follows:

- Model 1: The mean vacuum in b-phase should be constant at varying flow rates.
- Model 2: The mean vacuum in d-phase should be constant at varying flow rates.
- Model 3: The mean vacuum in both phases should show a considerable increasing trend, when the flow rate increases.

The criteria for the three models were chosen, because they represent three of the important and different adjustment possibilities for the teat-end vacuum in combination with the resulting massage pressure that meets the teat ends during milking with different “model settings”.

After selecting the vacuum data for the models related to the objectives mentioned above, the respective data settings for the flow rate and for the VCV settings were calculated from the characteristic curves and formulated as the control model. The three control models that were developed were the basis for the software production of the online vacuum control system. Finally, the functions for the adjustable mean vacuum were calculated and shown in a table for each model of the automatically controlled b-and d-phase vacuum. All the model functions were fitted to the given vacuum data with the help of the statistic software JMP 8.0 (SAS Institute).

To calculate the regression functions, the “Fit Model” function was used for models without control through 2. For Model 3 the nonlinear regression platform was used. Linear regression functions were used for the Model “without control” and Model 2. For Model 1, a polynomial function was fitted, while for Model 3 a logistic 5p-function was used and fitted by an iteration process, using a quasi-Newton BFGS optimization method. Starting parameters were derived visually by adjusting the five parameters with the slider function provided by JMP. The generalized logistic function is widely-used as a flexible sigmoid function for growth modelling. This function extends the possibilities of the well-known logistic curves [30]. For the variant without control a simple linear regression model of the form yi = μ + ax + εi was used. The influence of flow rate x on the vacuum level y, was then tested with an F-Test as part of an ANOVA model at a significance level of 0.05. Within the ANOVA, the parameters μ (intercept representing mean vacuum at flow rate of 0.0) and a (gradient) were tested for difference to zero with *t*-Tests.

## 3. Results

### 3.1. Characteristic Vacuum Curves and Vacuum Changes as a Function of Time

**Figure 3.**
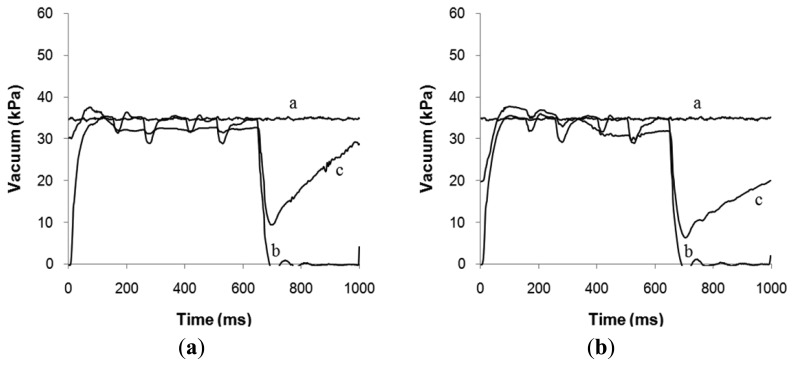
Vacuum changes as a function of time for Line-a: machine vacuum; Line-b: pulsation chamber vacuum; and Line-c: teat-end vacuum at flow rates of 0.5 (**a**) and 1.2 L/min per quarter (**b**) in MULTI, measured at the rear left quarter, without control [26].

In Figure 3 the main vacuum (line-a), the vacuum in the pulse tube (line-b) and the teat-end vacuum (line-c) is given as a function of time. For comparing the lowest point in (c) in both graphs in Figure 3, it was found that the minimum teat-end vacuum (c) is on moderate lower level in Figure 3(b) compared with Figure 3(a).

**Figure 4.**
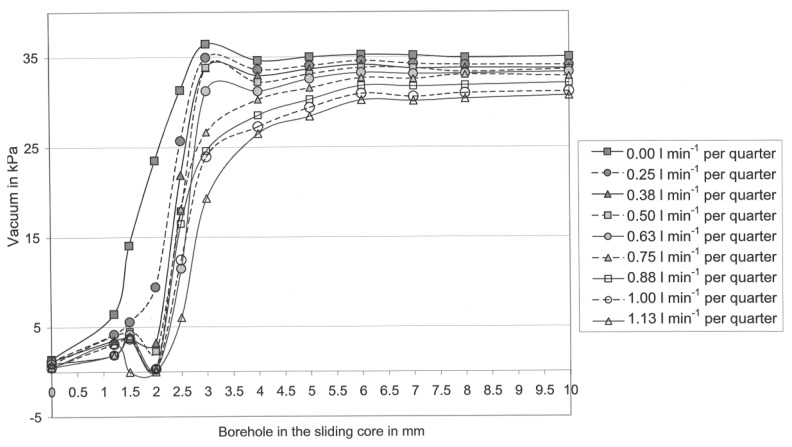
Characteristic curve of mean teat-end vacuum in b-phase depending on the boreholes in the sliding cores and on the liquid flow rate per udder quarter [26].

Figure 4 shows the effect of the diameter of boreholes in the sliding cores and the effect of the liquid flow-rate per udder quarter on the mean teat-end vacuum in b-phase.

**Figure 5.**
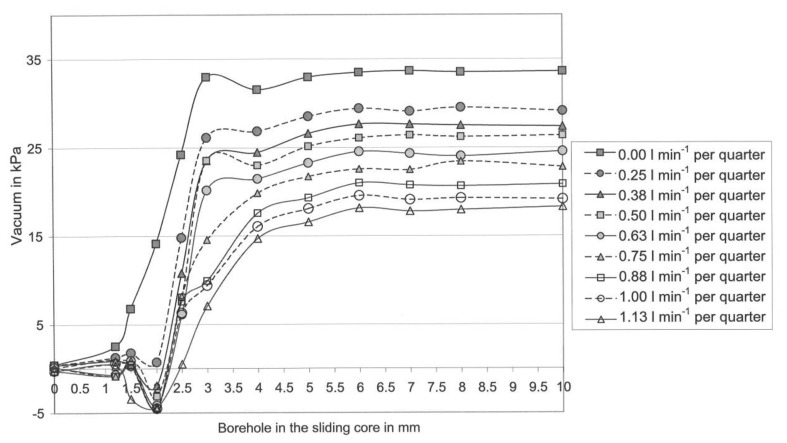
Characteristic curve of mean teat-end vacuum in d-phase, depending on the diameter of the boreholes in the sliding cores and depending on the liquid flow rate per udder quarter [26].

The highest mean vacuum in Figure 4 was 36.5 kPa and in Figure 5 it was 33.0 kPa. In both graphs, the mean teat-end vacuum (Sensor 3) subsides step-by-step with an increasing liquid flow rate per quarter.

A comparison of both graphs shows clearly that the vacuum in the d-phase in general, and especially at high flow rates, is much lower than in the b-phase. However, the higher the liquid flow, the stronger is the vacuum reduction effect in the Biomilker^®^ technology in the d-phase (Figure 5) [26]. This effect emerged at borehole sizes ranging between 2.5 and 10.0 mm in the sliding cores in VCV. Further, comparing Figures 4 with 5, it is visible that the teat-end vacuum remains constant with all tested boreholes with a diameter over 6.0 mm.

At all lower borehole diameters, with the exception of 3.0 mm and a shrinking in the borehole diameter has a vacuum-decreasing effect. At a borehole diameter of 3.0 mm, the vacuum reduction effect is lower. Observing all flow rates, it is obvious that the vacuum reduction effect begins in both pulsation phases with a borehole diameter of 6.0 mm.

### 3.2. The Three Control Models

**Figure 6.**
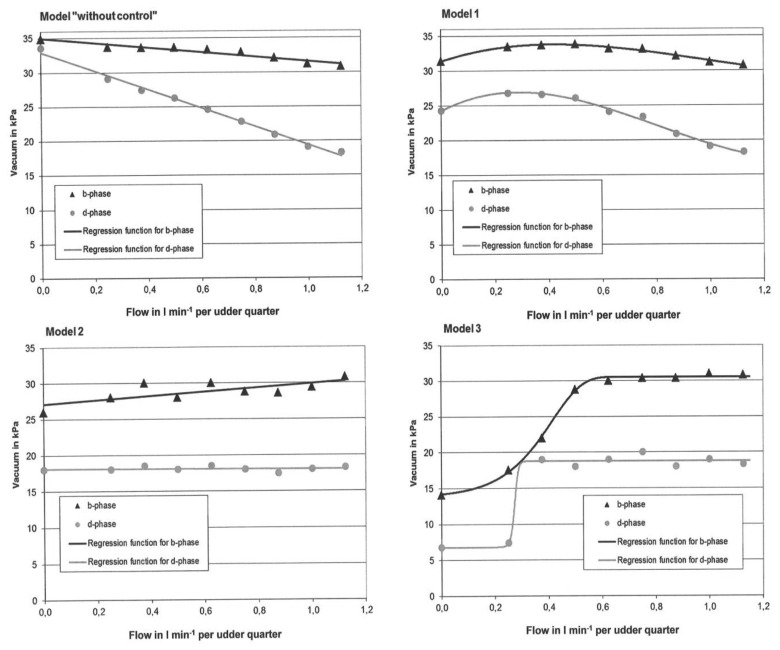
Vacuum behaviour in MULTI, depending on the flow rate per minute, per udder quarter, with the four possible models of the developed vacuum control system [26].

The three control models 1–3 that show a reaction to the teat-end vacuum conditions in the milking system are given in Table 1. Further, for these three models and for the reference Model “without control”, diagrams were generated and shown. The mean teat-end vacuum is shown, for the control models mentioned, as a function of the flow rate (Figure 6).

With the help of the three models of the simulated control system, it could be observed that the teat-end vacuum increases with an increasing flow rate. In Model 1 there is an increasing trend of the mean vacuum from a flow rate of 0.0 to 0.4 L/min per quarter. A decreasing trend was found at higher flow rates of 0.4 L/min per quarter. In Model 2, and especially in Model 3, there is a steadily rising trend visible for the teat-end vacuum over the whole range of possible flow rates. In contrast, it is typical of the Model “without control” (in comparison to models 1–3) that in the b-phase—and even more in the d-phase—that the teat-end vacuum falls significantly with an increasing milk flow. Especially at low flow rates under 0.5 L/min per quarter in b- and d-phases, the mean vacuum is much lower in all the models (1–3), compared to “without control” case. Among the three variations, Model 3 shows the lowest teat-end vacuum in both phases at low flow rates. Thus, the most important characteristic of the developed vacuum control system is that vacuum can be adjusted analogue, and not reverse to the flow rate of a milk flow curve. This is particularly true when Model 3 is switched on at the control system. The mathematical results for determining the model functions for all the eight curves (Figure 6) are given in Table 2.

### 3.3. Complete Vacuum Control System

A final result was the construction of the complete vacuum control system with sensing the milk flow via pressure sensors (described in [8]) and with an electric version of the VCV. The four VCVs in the complete vacuum control system are equipped with electric stepper motors for adjusting the opening space of the valve's outflow. This performed automatically during the milking process with a reaction rate with three- to ten-second reaction interval. A construction drawing of the electric VCV is given in the patent publication of Ströbel *et al.* [26].

**Figure 7.**
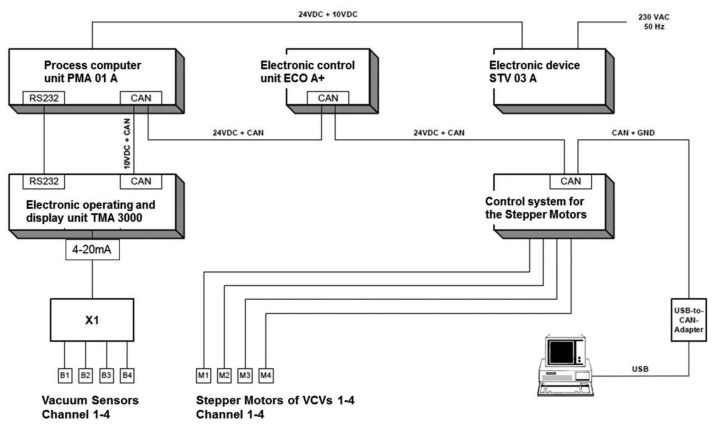
Block diagram for the vacuum control system. This system is fully integrated into the herd management system of the Impulsa AG (modified according to [32]).

The final vacuum control system was produced as a combined hardware and software solution by Impulsa AG (Elsterwerda, Germany) [31]. This was a result of the data from the developed model data in Table 2 of this publication. The final control system was programmed so that it would be possible to change the essential basic settings in the software, allowing for full adjustment of the control parameters to the specific device and the conditions in the milking parlour. The possibilities that were mentioned for changing the settings could be defined as the calibration of the vacuum control system. The block diagram for the finished vacuum control system is presented in Figure 7. The block diagram in Figure 7 presents all the required electric devices, which work together in combination and build the automatic vacuum control system.

## 4. Discussion

### 4.1. Characteristic Vacuum Curves

The highest teat-end vacuum for each flow rate has been found in nearly all cases in Figures 4 and 5, by using the sliding core with a borehole diameter of 10 mm. Furthermore, it was found for almost all cases that a decrease in the size of the borehole leads to a decrease in the teat-end vacuum. This points to a serious need for the technical implementation of the control system with computer-controlled valves and sensors. With that application, it is possible to exactly control the vacuum on the characteristic curve for each flow rate from 0.0 to 1.13 L/min per quarter.

However, for some vacuum values the relationship described is not true and these values were measured at borehole diameters between 1.2 and 3.0 mm. Further, it is noticeable that only in a few cases was unexpected turbulence visible, namely at borehole diameters between 1.0 and 2.0 mm (*cf*. Figures 4 and 5). This turbulence appears only in the timespan shortly before the whole milk flow is blocked. Thus, this kind of turbulence cannot disturb the control system in its effect on the teat-end vacuum control, because the control system should only be able to control the teat-end vacuum in limits between 7.5 and 35.0 kPa. At vacuum levels below approximately 7.5 kPa, the teat-cup would fall off [33]. Therefore, it can be stated that the borehole diameters with a size between 1.0 and 2.0 mm are too small for controlling purposes. Eventually the different liquid flow led to the turbulences especially at a borehole diameter of 3 mm.

O'Callaghan and Berry [34] investigated a conventional cluster (CON) and a self-developed individual quarter milking system (IQS) in a parlour with high line installation. A comparison of these data from the former study [34], with the data in Figures 4, 5 and 6 will show the potential effect of the control system as shown in Table 3. In the former study, the machine vacuum was 50 kPa for CON and IQS [34], what is a realistic level for high-line installations. MULTI works with the low level vacuum of 35 kPa and in low line installation. The milkline was 300 mm lower than the floor of the parlour, thus it was much lower than in the former study [34].

In Table 3 the mean teat-end vacuum reductions of CON and IQS are compared to the mean reductions of MULTI with VCV, set to Model 3 and MULTI without control and at the exemplary flow rate of 1.0 L/min per quarter. The MULTIs with the given two models in the present study have, in comparison to the single teat cup unit of O'Callaghan and Berry [34], clearly lower vacuum reductions in the b-phase of the pulse cycle (Table 3), with for example 3.5–4.0 kPa in MULTI in comparison to 17.0 kPa in IQS.

In the d-phase higher vacuum reductions can help to preserve the teat tissue [22,35,36]. The reduction level of MULTI leads to a mean vacuum level of 19.0 kPa in the d-phase. CON and IQS show a mean d-phase vacuum of 38.5 kPa and of 25.0 kPa. Thus, it can be stated that 38.5 kPa in the d-phase is surely a vacuum level that is too high for each kind of teat [36], measured in the laboratory by O'Callaghan and Berry [34]. Very high teat-end vacuum (for example about 39 kPa [9]) around the teat tissue has a negative effect on the udder health [3,10,22,35,36]. A vacuum level of 15.0 to 25.0 kPa seems to be an appropriate level for the protection of the teat tissue, especially when the liner is adapted to this level [36]. But the most important advantage of the MULTI, in combination with Model 3, is that the teat-end vacuum at high flow rates can be adjusted to a constant high mean (teat-end) vacuum for all flow rates higher than 0.6 L/min per quarter.

### 4.2. The Three Control Models

The developed vacuum control system is able to produce constant or steadily increasing teat-end vacuum at increasing flow rates. Moreover, Figure 6 shows that the hypothesis of that study is true for the b-phase in Model 2 and for the b- and d-phases in Model 3. For the other models the hypothesis is not true.

Further, Figures 4, 5 and 6 show that potentially the subject vacuum control system, offers a variety of different settings for controlling the teat-end vacuum individually for each udder quarter. By comparing the models with some state of the art milking systems, the difference in the teat-end vacuum reduction can be shown. A comparison of vacuum reductions in low line installations was made by Rose [25]. The Harmony plus milking cluster (CON), produced by DeLaval, (Tumba, Sweden) with 12.0 mm inner diameter short milk tubes, was compared with an individual quarter milking system (IQS) in the laboratory. Rose [25] found mean vacuum reductions (per pulse cycle) of approximately 13.8 kPa at a flow rate of 1.13 L/min per quarter for the conventional cluster (CON) and 3.5 kPa for IQS with four long milk tubes with a 16.0 mm inner diameter.

The three models show a lower teat-end vacuum reduction in comparison to CON and a higher reduction, compared with the IQS, at the presented flow rate in Figure 4. On the other hand, the reduction level of IQS is low, because it was equipped with tubes with a 16 mm inner diameter. However, these big tubes do not produce enough vacuum reduction in the d-phase, which is a disadvantage for this system [25]. Another fact shows that in Model 3 the vacuum reduction is almost constant for the flow rates ranging from 0.6 to 1.13 L/min per quarter. At this range, the CON in the former study shows an increase in the reduction of 4.0 kPa and in the IQS an increase of 1.0 kPa. The constant vacuum reduction of Model 3 in the b-phase at high flow rates is a significant advantage of this control model, because the vacuum can be adjusted much better on the quarter level, when the reduction level is constant and known over a wide range of flow rates.

In another study, Rose-Meierhöfer *et al.* [37] found a vacuum reduction in the b-phase of 10.4 kPa at a flow rate of 1.13 L/min per quarter in milking-time tests for a CON (GEA Classic 300, GEA Farm Technology, Bönen, Germany) with a claw volume of 300 cm^3^ at a machine vacuum of 42 kPa. The reduction of 10.4 kPa was compared to the results of the controlled models (models 1–3). This shows a reduction of only 4.0 kPa after both had been measured in the b-phase, at the same flow rate. Thereby, it can be observed that the control system can help to avoid vacuum reductions. For the b-phase, it is true that a high vacuum level, which happens especially at high milk flow rates, leads to teat cups falling off and to a delay in the milking time of cows [38–40].

Another argument for the usefulness of the control system is that low flow rates do not require a high vacuum for the transportation of the milk to the receiver. Thus, a high vacuum at the lower flow rates (under 0.5 L/min per quarter) is redundant. Besides, it only attacks the teat tissue, especially at the end and partially at the beginning of a milking process. A further advantage is that over-milking can be reduced by using this new system. When the control system is introduced, over-milking can occur only when the teat-end vacuum is strongly reduced. Further, currently available switch-off functions for avoiding over-milking do not operate as frequently during the whole milking process and they can remove the milking cluster only when the slowest quarter is properly milked out. Thus, up to three teats are charged with a vacuum that is too high at the end of the milking process.

Further, Öz *et al.* [41] showed that there were vacuum reductions at a flow rate of 0.2 L/min per quarter in the d-phase, of 2.0 kPa in the conventional system, of 3.3 kPa in the Biomilker^®^ cluster, and of 8.0 kPa in the Multilactor^®^ system. With the control system (Figure 6), the reductions in the d-phase at the same flow rate are 8.0 kPa (Model 1), 16.9 kPa (Model 2) and 28.0 kPa (Model 3). Thus, the force on the teats at low flow rates is much lower when the control system is used. This is also true in comparison to teat-preserving milking technologies which have a high vacuum reduction in d-phase like System Happel^®^ or Biomilker^®^. To get earlier results, it is important to consider on farm, which of the three models is the optimal for the teat-end and for an efficient milk production.

Thus, Reinemann *et al.* [39] argue that low milking vacuum has a positive effect on the teat condition but a negative one on the milking time. In addition, a longer milking time increases the risk of mastitis, as the teat cups have longer contact with the teat. Model 3 has been selected as the best control model, because the effect on the teat-end vacuum meet the requirements for desirable teat-end vacuum better than the other two control models 1 and 2. In Model 3 the vacuum is kept low most consistently in the phases, where no increase of the milking time through low vacuum is expected, particularly in the d-phase and during milking flows under 0.5 L/min per quarter. In Model 2 the vacuum in the d-phase was kept constant. That also ensures an optimal rest of the teat during a potential over-milking or low liquid flow time. But even here the implementation of Model 3 is more consistent. The above mentioned arguments exclude Model 1, in which the vacuum in the b- and d-phases is still too high for flow rates from 0.0–0.9 L/min per quarter. Throughout the whole d-phase a low vacuum is desirable, as is a massage effect to the teats.

### 4.3. Vacuum Control and Vacuum Stability for Dairy Cows and for Other Dairy Species

In a study of Ambord and Bruckmaier [42], a milking system with partial compensation of flow-related vacuum was compared with a standard, (high-line) milking system for cows. The peak milk flow rate was higher and the plateau milking duration was lower as in the standard milking system. Total milk time, duration of incline and decline phase and average milk flow did not differ in the study [42].

The study could show that the peak milk flow was higher, when the vacuum reduction caused by high milk flows was compensated. The vacuum control system in the current study has in total a similar effect of guaranteeing higher vacuum a high flow rates, but also focus on vacuum reduction at low milk flow rates. Further, in the study of Ambord and Bruckmaier [42], an effect of the increased vacuum stability on teat condition and udder health could not be found, but the experiment took time only 7 days per group with 10 cows. Thus, it would be interesting to test the vacuum control system of the current study with the methods of the cited study [42], for a longer period of time, to observe the effect of vacuum reduction low milk flow rates on teat condition and udder health.

Another study of Caria *et al.* [43] tested the effect of six different machine vacuum levels (range 37–52 kPa) on general milking and milking time of Italian buffalo. One result of the study was that lower machine vacuum result in a decrease in average and peak milk flow. That is negative. Another result was that a lower machine vacuum between 37 and 40 kPa led to a better ratio between plateau and decline phase in the milk flow curves, without relevant time lag for the total milking time. In general the authors [43] advised to milk buffalos with the stated lower machine vacuum. Before, Caria *et al.* [44] found in a comparison between adjustment of 36 kPa and 42 kPa machine vacuum for buffalo, that a vacuum of 36 kPa led to an increase of milking time, time lag and decreased the average flow rate.

The developed control system of the current study, can help to find the optimum teat-end vacuum during farm tests and can adjust the found vacuum for each herd (or even for each quarter of a cow or buffalo) individually. Thus, high and low teat-end vacuum can be adjusted, when required by the animal. Positive effects of optimal vacuum can be maximised by adjusting teat-end and machine vacuum.

To focus on the most important results of the study, it was found that Model 3 for teat-end vacuum adjustment is at the moment, the best alternative for controlling the teat-end vacuum. The vacuum control system developed in this study can probably be generated with software that includes all three control models, *i.e.*, 1–3. Therefore, the effect of the control models on the teat condition, cell count and machine-on-time should be soon tested empirically. In the future, the vacuum reduction as a parameter for evaluating milking systems will lose its importance on account of the findings presented in this paper. It is better to check the b- and d-phase teat-end vacuum curve of each milking system and each single milking in detail and to search for parameters which will help to evaluate the quality of the vacuum application in a better way. For sure, it will be possible in the future to find standard parameters for the teat-end vacuum as a function of the flow rate and the morphological teat type, which can be added to the ISO-guideline [24].

## 5. Conclusions

The present study leads to the following conclusions:

- All three control models (1–3) produce liquid-flow-related data for the teat-end vacuum in comparison to the Model “without control”.
- Model 3 has been selected as the best control model, because the effect on the teat-end vacuum meet the requirements for desirable teat-end vacuum better than the other two control Models 1 and 2.
- The target function showing the effect of the liquid flow (x) on the teat-end vacuum (y) in the selected Model 3 has the formula: y = (A) + (B − A)/[(1 + exp(C + Dx))^E^].
- It is possible to produce a constant or increasing mean teat-end vacuum in the b- and d-phases at varying flow rates, with the vacuum control system presented here.
- The control system can be introduced to all kinds of individual quarter milking systems and these systems are increasing their market share continuously at the moment.
- The models of the control system should be investigated in farm experiments to find out which kinds of settings affect the udder health of various cows.

## Figures and Tables

**Table 1.** Three developed control models 1–3 for controlling the teat-end vacuum with different desired effects on the vacuum [26].

**Model 1 Milk Flow Intervals (mL/min/quarter)**	**Borehole Diameter in VCV (mm)**	**Model 2 Milk Flow Intervals (mL/min/quarter)**	**Borehole Diameter in VCV (mm)**	**Model 3 Milk Flow Intervals (mL/min/quarter)**	**Borehole Diameter in VCV (mm)**
0 ≤ X ≤ 125	2.5	0 ≤ X ≤ 125	2.2	0 ≤ X ≤ 125	1.5
125 < X ≤ 313	4.0	125< X ≤ 313	2.6	125 < X ≤ 313	2.3
313 < X ≤ 438	5.0	313 < X ≤ 563	2.7	313 < X ≤ 438	2.5
438 < X ≤ 563	6.0	563 < X ≤ 688	2.9	438 < X ≤ 563	2.7
563 < X ≤ 688	7.0	68 8< X ≤ 813	3.5	563 < X ≤ 688	2.9
688 < X ≤ 813	8.0	813 < X ≤ 938	4.0	688 < X ≤ 813	4.0
813 < X ≤ 2,500	10.0	938 < X ≤ 1,063	5.0	813 < X ≤ 938	5.0
		1,063 < X ≤ 2,500	10.0	938 < X ≤ 1,063	6.0
				1,063 < X ≤ 2,500	10.0

**Table 2.** Regression functions and model parameter estimates for all the data given in Figure 6.

**Model**	**Without Control (Linear Regression)**		**1 (Cubic Regression)**	
Function	y = Dx + E		y = Bx^3^ + Cx^2^ +Dx + E	
Phase	b	d	b	D
Estimate ± StdErr				
A	–	–	–	–
B	–	–	6.63 ± 1.75 (*)	17.51 ± 3.60 (*)
C	–	–	−7.03 ± 0.60 (*)	−10.16 ± 1.23 (*)
D	−3.43 ± 0.36 (*)	−13.54 ± 0.43 (*)	−3.44 ± 0.44 (*)	−12.12 ± 0.90 (*)
E	35.00 ± 0.26 (*)	32.95 ± 0.30 (*)	35.52 ± 0.27 (*)	32.05 ± 0.56 (*)
Adj. R^2^	0.916	0.992	0.974	0.985
RMSE	0.378	0.441	0.187	0.384
**Model**	**2 (Linear Regression)**		**3 (5-Parametric Richards Function)**	

Function	y = Dx + E		y = (A) + (B − A)/((1 + exp(C + Dx)) ^E^)	
Phase	b	d	b	D
Estimate ± StdErr				
A	–	–	30.54 ± 0.20	18.76 ± 0.32
B	–	–	13.79 ± 0.71	6.80 ± 0.78
C	–	–	−10.51 ± 498.15	−33.57 ± 129878.54
D	2.81 ± 1.02 (*)	−0.08 ± 0.32	8.95 ± 2.91	120.98 ± 0.00
E	27.12 ± 0.72 (*)	18.14 ± 0.22 (*)	894.80 ± 446491.67	1.45 ± 185526.47
Adj. R^2^	0.452	−0.132	–	–
RMSE	1.060	0.330	0.440	0.784

Where: y = Vacuum in kPa; x = Flow rate in L/min per udder quarter; StdErr = Standard Error; (*) = distinguish significant from 0, when α = 0.05; Adj. R^2^ = Adjusted coefficient of determination and RMSE = root mean square error.

**Table 3.** Comparison of vacuum reduction data in kPa and in (%) of machine vacuum of a CON and an IQS milking system taken from a study by O'Callaghan and Berry [34], with the data of MULTI, with and without control system, collected for the present study.

**Phase of Pulse**	**Flow Rate in L/min/quarter**	**CON**	**IQS**	**MULTI With-out Control**	**MULTI Model 3**
b-phase	1.0	6.2 [12.4]	17.0 [34.0]	3.5 [10.0]	4.0 [11.4]
d-phase	1.0	11.5 [23.0]	25.0 [50.0]	16.0 [45.7]	16.0 [45.7]
